# Genome Sequence of “*Candidatus* Serratia symbiotica” Strain IS, a Facultative Bacterial Symbiont of the Pea Aphid *Acyrthosiphon pisum*

**DOI:** 10.1128/MRA.00272-19

**Published:** 2019-05-09

**Authors:** Naruo Nikoh, Ryuichi Koga, Kenshiro Oshima, Masahira Hattori, Takema Fukatsu

**Affiliations:** a Department of Liberal Arts, The Open University of Japan, Chiba, Japan; b Bioproduction Research Institute, National Institute of Advanced Industrial Science and Technology, Tsukuba, Japan; c Center for Omics and Bioinformatics, Graduate School of Frontier Sciences, University of Tokyo, Kashiwa, Japan; d Department of Biological Sciences, Graduate School of Science, University of Tokyo, Tokyo, Japan; e Graduate School of Life and Environmental Sciences, University of Tsukuba, Tsukuba, Japan; Indiana University, Bloomington

## Abstract

“*Candidatus* Serratia symbiotica” is a facultative bacterial symbiont of aphids that affects various ecological traits of the host insects. Here, we report the complete genome sequence of “*Candidatus* Serratia symbiotica” strain IS, consisting of a 2,736,352-bp chromosome and an 82,605-bp plasmid, from the pea aphid Acyrthosiphon pisum.

## ANNOUNCEMENT

The candidate species “*Candidatus* Serratia symbiotica” represents a bacterial clade of facultative symbionts associated with the pea aphid Acyrthosiphon pisum and other aphid species, and it belongs to the Enterobacteriaceae family of the *Gammaproteobacteria* (1). The symbiont exhibits localization to secondary bacteriocytes, sheath cells, and the hemolymph of the host aphids (2–4), and it has been detected in populations of A. pisum across the world (5–8). Previous studies revealed that infection by the symbiont entails various context-dependent fitness benefits on the host aphid, such as tolerance to heat stress (9, 10), resistance to parasitoid wasps (11), partial complementation for loss of the essential symbiont Buchnera aphidicola (3, 12), and others.

Here, we analyzed the genome of “*Ca.* Serratia symbiotica” strain IS, a facultative symbiont of *A. pisum* that was reported to be capable of partially rescuing the absence of Buchnera aphidicola (3, 12). We collected body fluid from *A. pisum* strain AIST^IS^, which was generated by artificial infection of the symbiont from its original host aphid strain, because the transfected aphid strain exhibited higher bacterial density than did the original aphid strain (2, 3). Surface-sterilized adult aphids were dissected and washed in phosphate-buffered saline, the fluid was collected and filtered through 100-μm, 50-μm, 10-μm, and 5-μm nylon meshes, and the filtrate was subjected to DNA preparation. Genomic DNA was extracted by a standard phenol-chloroform method. The DNA sample (about 10 μg) was sheared to generate DNA fragments of 2 to 4 kb, ligated to the pUC18 vector for shotgun library construction, and subjected to Sanger sequencing using a fluorescent dye terminator method (13). Of the 30,801 sequence reads determined, 741 reads were assigned to aphid genes (14), whereas 74 reads were attributed to *B. aphidicola* genes (15). The remaining 29,986 reads were subjected to assembly using the Phred (v0.020425.c)-Phrap (v1.080812)-Consed (v20.0) package (16) and gap filling, as described previously (13), which finally yielded a circular bacterial chromosome (2,736,352-bp genome, 52.1% GC content) and a circular plasmid (82,605 bp, 44.8% GC content). We assessed the quality of the finished sequence by the Phred score (≥40). Putative protein-coding sequences (CDSs), tRNAs, and other noncoding RNAs were identified using GLIMMER 3.0 (17), tRNAscan-SE2.0 (18), and Rfam (19), respectively. The annotation of CDSs was based on BLASTP searches against UniProt (20). Default parameters were used in these analyses. The chromosome contained 2,001 putative protein-coding genes (of which 133 were located within repetitive sequences as transposases, etc.), 21 rRNA genes, 60 tRNA genes, and 651 pseudogenes. The plasmid carried 93 protein-coding genes, of which 38 were related to conjugation or DNA/protein transport to the host cell.

Genome sequences of “*Ca.* Serratia symbiotica” have been analyzed for another *A. pisum*-associated strain, Tucson, involved in the host’s tolerance to heat stress (21), strain CWBI-2.3^T^, isolated from the black bean aphid Aphis fabae (22), strain SCt-VLC from the cypress pine aphid Cinara tujafilina (23), the genome-reduced strain SCc from the cedar aphid Cinara cedri (24), another genome-reduced strain, SCifornacula, from the green spruce aphid Cinara fornacula (25), and a highly genome-reduced strain, STs, from the giant willow aphid Tuberolachnus salignus (26). Our new genome sequence will provide further insight into the biology, evolution, and diversification of the symbiont clade “*Ca.* Serratia symbiotica.”

### Data availability.

The genome sequence data for “*Ca.* Serratia symbiotica” strain IS have been deposited in the DNA Data Bank of Japan under accession no. AP019531 (chromosome), AP019532 (plasmid), and DRA008151 (raw sequence reads).
